# The Role of Amnioreduction in Emergency Cervical Cerclage with Bulging Membranes: A Retrospective Comparative Study

**DOI:** 10.3389/fsurg.2022.928322

**Published:** 2022-07-12

**Authors:** Yuan Zhang, Qingqing Wang, Zhangmin Tan, Jin Zhou, Peizhen Zhang, Hongying Hou, Yuzhu Yin, Zhenyan Han

**Affiliations:** Department of Obstetrics and Gynecology, Third Affiliated Hospital of Sun Yat-sen University, Guangzhou, China

**Keywords:** emergency cervical cerclage, prolongation of pregnancy, amnioreduction, cervical dilation, bulging membranes

## Abstract

The purpose of this study was to investigate the role of amnioreduction in patients who underwent emergency cervical cerclage (ECC) with bulging membranes during the second trimester. This retrospective comparative study included 46 singleton pregnant women who had cervical dilation at least 1 cm with bulging membranes beyond the external cervical os and underwent ECC at the Third Affiliated Hospital of Sun Yat-sen University between December 2016 and December 2021. Cases were categorized as amnioreduction group (*n* = 16) and non-amnioreduction group (*n* = 30) according to whether amnioreduction was performed prior to ECC. The gestational age and cervical dilation at cerclage, operative time, prolongation of pregnancy, and outcomes of pregnancy were compared between the two groups. All 46 patients underwent successful ECC excepted one case with intraoperative rupture of membrane in non-amnioreduction group. In the amnioreduction group, the cervical dilation at cerclage was larger than that in the non-amnioreduction group (4.5 ± 2.2 vs. 2.2 ± 1.2 cm, *P* < 0.001), and had more patients with cervical dilation ≥4 cm (50.0% vs. 10.0%, *P* = 0.004). However, the gestational age at cerclage, operative time, prolongation of pregnancy, gestational age at delivery were not significantly different between the two groups (22.9 ± 2.8 vs. 22.9 ± 3.2 weeks, 31.1 ± 9.2 vs. 27.9 ± 11.4 min, 21.3 ± 21.5 vs. 38.7 ± 40.2 days, 25.9 ± 4.5 vs. 28.4 ± 6.1 weeks; *P* > 0.05). The rates of delivery ≥28 weeks, ≥32 weeks, and live birth were 20.0% vs. 80.0%, 12.5% vs. 26.7%, 56.3% vs. 66.7% (*P* > 0.05) in amnioreduction group and non-amnioreduction group, respectively. In conclusion, even in cases with larger cervical dilation, the application of amnioreduction with ECC is possible to get the acceptable pregnancy outcomes. These findings suggested that amnioreduction may be a safe and feasible option to be applied before ECC, especially for those with advanced cervical dilation and bulging membranes.

## Introduction

The loss of mid-term pregnancy and premature birth are major problems in obstetrical fields, which brings great burden to the society and family [1]. Cervical incompetence is a status of pregnancy in which the cervix begins to dilate and efface before the term of pregnancy [2, 3]. It has been shown that the rate of preterm birth before 37 gestational weeks in patients with cervical incompetence is 3.3 times higher than those without cervical incompetence, accounting for 8–9% of all preterm births [4]. The live birth rate is estimated to be 23% at 23 weeks of gestation, 38% at 24 weeks of gestation and 54% at 25 weeks of gestation [5, 6], suggesting that prolonging gestational age can significantly improve the live birth rate of premature infants. According to the ACOG (American College of Obstetricians and Gynecologists) guideline on cerclage for the management of cervical insufficiency, the diagnosis of cervical insufficiency mainly depends on the past medical history [3]. For women with a history of cervical insufficiency, preventive cervical cerclage is an effective surgical method for treating cervical insufficiency, which can effectively prolong the gestational age and reduce the adverse perinatal outcomes[3, 7–10]. However, for women with the first pregnancy, or those without a history of mid-trimester pregnancy loss or spontaneous preterm birth, emergency cervical cerclage (ECC) is a salvage procedure when cervical dilation with bulging fetal membranes but no signs of labor and infection in mid-trimester, in an attempt to prolong the pregnancy to a viable gestation [11–14]. However, compared to the elective cervical cerclage, ECC is a technically challenging procedure, as the protruding membranes in the cervix making it difficult to place the cervical suture properly and carry the risk of iatrogenic rupture of the membranes during surgery [11–13, 15]. In addition, ECC is also associated with worse pregnancy outcomes including the earlier gestation age at delivery, lower birth weight, lower live birth rate, and higher intra-amniotic infection rate, especially for women with advanced cervical dilation [11, 16–20]. Moreover, ECC is not always successful in all patients with cervical dilation, especially when dilation is completely [3, 7, 10, 14].

In 1979, Robert C. Goodwin reported for the first time the application of amnioreduction before ECC to reduce the tension of the protruding amniotic sac in the vagina [21]. Since then, several studies have reported on amnioreduction prior to ECC for bulging membranes, however, the pregnancy outcomes were inconsistent due to the heterogeneity of cases [22–26]. To improve the success of ECC procedure and pregnancy outcomes among patients with large bulging membranes, our hospital started to adopt amnioreduction prior to ECC since December 2016. In this study, we retrospectively compared the clinical features and pregnancy outcomes of patients who underwent ECC with bulging membranes between 16 cases with amnioreduction and 30 cases without amnioreduction and evaluated the safety and effectiveness of amnioreduction in ECC during the second trimester.

## Methods

### Study Design and Subjects

This retrospective cohort study recruited pregnant women who had painless cervical dilation with bulging membranes beyond the external cervical os and underwent ECC during the second trimester at the Third Affiliated Hospital of Sun Yat-sen University between December 2016 and December 2021. Inclusion criteria were: (1) singleton pregnancy. (2) gestational age between 14^+0^ and 27^+6^ weeks. (3) cervical dilatation of at least 1 cm and bulging membranes were confirmed by transvaginal ultrasound and speculum examination. (4) ECC were performed within 24 h after cervical dilation diagnosed, not waiting for the results of vaginal microbiotic smear. Exclusion criteria included: (1) premature rupture of membranes. (2) persistent or active vaginal bleeding (3) regular uterine contractions that occurred before operation. (4) clinical chorioamnionitis (maternal body temperature ≥38°C, with fetal heart rate ≥160 beats/min or maternal peripheral blood leukocyte count ≥15 × 10^9^/L. (5) severe pregnancy complications and disease. (6) fetal structural abnormalities.

From December 2016, amnioreduction was applied prior to ECC at our hospital when cervical dilation is ≥3 cm or difficult to replace the bulging membranes unless the patients refuse amnioreduction, or the deepest vertical pocket of amniotic fluid is less than 30 mm, or it is difficult to avoid puncturing placenta. This study was approved by the institutional review board of the Third Affiliated Hospital of Sun Yat-sen University and written informed consent was obtained from each patient.

### ECC and Amnioreduction

At admission, after extensive counseling on the potential risk of miscarriage and failure of the operation, all pregnant women had signed informed consent for ECC with or without amnioreduction. The patients were placed in a lithotomy position with steep Trendelenburg tilt under spinal anesthesia. Vaginal speculum examination reconfirmed the cervical dilation and the tension of bulging membranes. The protruding membranes were gradually retracted into the uterus by inflated Foley catheter or cervical cook balloon, or moistened gauze ball according to the size of cervical dilation and discretion of operator. Cervical cerclage was performed using the McDonald techniques. If the cervical dilation is larger than 3 cm or prolapsed amniotic sac is hard to repositioned, a transabdominal amnioreduction would be needed before ECC unless the placental penetration could not be avoided. After emptying the bladder, ultrasound-guided freehand techniques were conducted with 22G needle punctured into the amniotic cavity. The amount of amniotic fluid reduction was determined by the distension of the bulging sac, usually from 50 to 250 ml. If having the indication of prenatal diagnosis, the obtained amniotic fluid was sent for fetal chromosomal karyotyping with or without microarray analysis under the consent of the pregnant women.

### Perioperative and Postoperative Treatment

During the perioperative period, all the patients were given treatment regimen consisting of five schemes, included: bed rest until 34 gestational weeks, intravenous broad-spectrum antibiotics (cephalosporins or azithromycin + metronidazole) for 5 to 7 days after the operation, tocolytics (including indomethacin + ritodrine hydrochloride, or atosiban) for at least 48 h, prophylactic venous thromboembolism with low molecular weight heparin until 34 gestational weeks with normal physical activity or presenting the following indication for removal sutures, and dexamethasone for fetal lung maturation (after 24 gestational weeks). However, the specific course of tocolytic treatment was individualized at the discretion of senior obstetrician, mainly based on uterine contractions, gestational age and whether presenting clinical chorioamnionitis, because there was no uniformity and guideline on this issue.

Cerclages would be removed around 37 weeks of gestation or presenting the indication for earlier removal sutures such as active preterm labor, clinical evidence of chorioamnionitis, heavy vaginal bleeding, preterm premature rupture of membranes, or fetal compromise.

### Data Collection

Maternal data included age, previous obstetrics history, prior cervical intervention, gestational weeks, ultrasound examination, size of cervical dilation, preoperative white blood cell count, C-reactive protein level, vaginal microbiotic smear, details of the operation, and perioperative management. Maternal and fetal outcomes included prolongation of pregnancy, gestational weeks at delivery, mode of delivery, complications, birth weight, perinatal death or survival, and placental pathologic examination.

### Statistical Analysis

SPSS statistical software version 26.0 was used to analyze the data. Shapiro–Wilk was used to test the normality of continuous variables. According to the characteristics of the variables, data are presented as means ± standard deviations (SD) and median (interquartile range) for continuous variables and numbers (percentages) for categorical variables, respectively. The differences were compared between groups using Student's *t*-test (Gaussian distribution data) and Mann-Whitney *U* test (non-Gaussian distribution data). Chi-squared test or Fisher's exact test was used to analyze the categorical variables. *P* value of <0.05 was considered statistically significant. G*power 3 software [27] was used to perform the sample size calculation, and the effective size was calculated based on the present results with a significance level of 0.05 (one-tailed) and a power of 0.8.

## Results

During the study period, a total of 126 pregnant women who had short cervix or cervical dilation underwent ECC. Among 46 singleton pregnancies with cervical dilation and bulging membranes, 16 cases accepted ECC with amnioreduction and 30 cases that accepted ECC without amnioreduction were eligible for amnioreduction group and non-amnioreduction group, respectively (Supplemental Table S1 and S2). All 46 patients underwent successful ECC except one case with intraoperative membrane rupture in the non-amnioreduction group. The results of fetal chromosomal karyotyping and microarray analysis of amniotic fluid from patients with advanced maternal age or abnormal biochemical screening were normal.

### Clinical Characteristics of Included Patients Before the Operation

The mean maternal age in amnioreduction group was older than that in the non-amnioreduction group (32.5 ± 4.4 vs. 29.4 ± 4.3 years, *P* = 0.016). The two groups were similar in the mean gestational age at cerclage, rates of primigravidity and nulliparous, rate of previous spontaneous miscarriage during the first and the second trimester, white blood cell count before the operation, and preoperative C-reactive protein value (Table 1). However, the preoperative size of cervical dilation in the amnioreduction group was significantly larger than that in the non-amnioreduction group (4.5 ± 2.2 vs. 2.2 ± 1.2 cm, *P* < 0.001), and had more patients with cervical dilation ≥4 cm (50.0% vs. 10.0%, *P* = 0.004) (Table 1).

**Table 1:** Baseline clinical characteristics of the included patients.

Variables	Amnioreduction group (*n* = 16)	Non-amnioreduction group (*n* = 30)	*P* value
Maternal age (years)	32.5 ± 4.4^e^	29.4 ± 4.3^e^	0.016^a^
Gestational age at cerclage (weeks)	22.9 ± 2.8^e^	22.9 ± 3.2^e^	0.993^a^
Primigravidity, *n* (%)	7 (43.8%)	9 (30.0%)	0.351^b^
Nulliparous, *n* (%)	12 (75.0%)	19 (63.3%)	0.421^b^
Previous spontaneous miscarriage during first trimester, *n* (%)	4 (25.0%)	7 (23.3%)	0.585^c^
Previous spontaneous miscarriage during second trimester, *n* (%)	1 (6.3%)	5 (16.7%)	0.307^c^
Cervical dilatation before operation (cm)	4.5 ± 2.2	2.2 ± 1.2	<0.001^d^
	4.0 (3.3)	2.0 (2.0)	
Cervical dilatation before operation ≥4 cm, *n* (%)	8 (50.0%)	3 (10.0%)	0.004^c^
White blood cell count before operation (×10^9^/l)	14.6 ± 8.0^e^	12.7 ± 3.0^e^	0.960^d^
	11.7 (4.9)^f^	12.8 (3.3)^f^	
C-reactive protein value before operation (mg/l)	10.9 ± 5.3^e^	11.6 ± 9.1^e^	0.702^d^
	10.2 (9.0)^f^	8.0 (12.2)^f^	

^a^ *Student's t-test*.

^b^ *chi-squared test*.

^c^ *Fisher's exact test*.

^d^ *Mann Whitney U test*.

^e^ *means ± standard deviations*.

^f^ *median (interquartile range)*.

### Outcomes After the Operation

The pregnancy outcomes were presented in Table 2, Supplementary Table S1 and Supplementary Table S2. There were no significant differences when comparing the amnioreduction group and non-amnioreduction group in terms of operative time, prolongation of pregnancy, gestational age at delivery, rates of delivery ≥28 weeks, delivery ≥32 weeks, preterm premature rupture of membranes, clinical chorioamnionitis, and acute histologic chorioamnionitis. The neonatal outcomes were also statistically comparable in terms of birthweight (≥24 weeks), 1 min Apgar scores, 5 min Apgar scores, and the total survival rate between the amnioreduction group and non-amnioreduction group. There was no maternal morbidity related to ECC that occurred from operation to postdelivery.

**Table 2:** Clinical outcomes after surgery between amnioreduction group and non- amnioreduction group.

Variables	Amnioreduction group (*n* = 16)	Non-amnioreduction group (*n* = 30)	*P* value
Operative time (minutes)	31.1 ± 9.2^e^	27.9 ± 11.4^e^	0.179^a^
	30.0 (15.3)^f^	26.5 (10.5)^f^	
Prolongation of pregnancy (days)	21.3 ± 21.5^e^	38.7 ± 40.2^e^	0.221^a^
	14.5 (21.8)^f^	26.5 (53.8)^f^	
Gestational age at delivery (weeks)	25.9 ± 4.5^e^	28.4 ± 6.1^e^	0.159^b^
Preterm premature rupture of membranes, *n* (%)	5 (31.3%)	11 (36.7%)	0.713^c^
Clinical chorioamnionitis, *n* (%)	2 (12.5%)	1 (3.3%)	0.274^d^
Acute histologic chorioamnionitis, *n* (%)	9/13 (69.2%)	13/22 (59.1%)	0.409^d^
Miscarriage or abortion < 24 weeks, *n* (%)	6 (37.5%)	7 (23.3%)	0.248^d^
Delivery 24-27^+6^ weeks, *n* (%)	6 (37.5%)	7 (23.3%)	0.248^d^
Delivery ≥28 weeks, *n* (%)	4 (20.0%)	16 (80.0%)	0.065^c^
Delivery ≥32 weeks, *n* (%)	2 (12.5%)	8 (26.7%)	0.236^d^
Neonatal birthweight (≥ 24 weeks, g)	1267 ± 503^e^	1683 ± 932^e^	0.196^a^
	1135 (777.5)^f^	1350 (1200)^f^	
Live birth, *n* (%)	9 (56.3%)	20 (66.7%)	0.486^c^
Neonatal birthweight (g)	1331 ± 487^e^	1843 ± 894^e^	0.140^a^
	1150 (715)^f^	1575 (1450)^f^	
1-min Apgar score	8.8 ± 1.9^e^	9.2 ± 1.6^e^	0.317^a^
	9.0 (1.5)^f^	10.0 (1.5)^f^	
5-min Apgar score	9.6 ± 0.5^e^	10.0 ± 0.2^e^	0.095^a^
	10.0 (1.0)^f^	10.0 (0.0)^f^	
Neonatal survival, *n* (%)	9 /9 (100.0%)	20/20 (100.0%)	/

^a^ *Mann Whitney U test*.

^b^ *Student's t-test*.

^c^ *chi-squared test*.

^d^ *Fisher's exact test*.

^e^ *means ± standard deviations*.

^f^ *median (interquartile range)*.

## Discussion

In this retrospective cohort study, we compared the clinical features and pregnancy outcomes from singleton patients who had cervical dilation and bulging membranes and underwent ECC with or without amnioreduction. The results showed that there were statistically comparable prolongation of pregnancy and perinatal outcomes among the two groups, even women with amnioreduction had larger cervical dilation, suggesting that amnioreduction may be a safe and feasible option to be applied before ECC, especially for those with advanced cervical dilation and bulging membranes.

As a salvage procedure, patients eligible for ECC all have cervical dilation with or without protruding membranes before the operation [3, 7, 10]. Therefore, the difficulty of performing ECC is higher than the standard procedure of cervical cerclage. When the cervical external os is open, the inferior amniotic sac would prolapse through the cervical canal or even protruding into the vagina. The membranes bulging outside the cervix will elevate the distension of prolapsed sac, and exposure to vaginal bacteria would increase the risk of infection [23, 28–30]. In addition, repeatedly pushing the membranes during surgery will further elevate the distension of the sac, thus increasing the risk of membranes rupture and uterine contraction. Therefore, successfully replacing the bulging membranes into the uterus is the key step for ECC. Some devices including moist gauze and inflated balloon (Foley catheter, cervical cook balloon, uniconcave balloon and balloon tamponade) have been used during ECC [11, 14, 31, 32]. However, for women with advanced cervical dilation or increased pressure of intra-amniotic sac, the above devices may not be effective due to limited contact area with membranes, limited supporting force or risk of rupture of membranes. In our series, the bulging membranes from eight cases with cervical dilation at 2–3 cm were hard to repositioned into the uterine cavity by moist gauze or inflated balloon due to the higher intra-amniotic pressure, resulting rupture of the membrane during the procedure in one case and turn to amnioreduction in seven cases.

Several studies demonstrated that patients with cervical dilation of more than 3–4 cm and bulging membranes had higher chance of failure of ECC, short prolongation time and more pregnancy complications [11, 14, 20, 33]. Some authors even concluded that ECC was not a rational option when patients had cervical dilation of more than 4 cm and protruding membranes [20]. However, a study by Pereira L. et al. demonstrated that if underwent expectantly management, women presenting with advanced cervical dilation of more than 4 cm would ultimately deliver within one week [34]. The main purpose of amnioreduction prior to ECC is to reduce intrauterine pressure. After amnioreduction, it can be observed that the distension of the membranes was markedly reduced and makes it easier to expose dilated cervical edge, in turn reducing the surgical difficulty and improving outcomes [21, 23–26]. In our study, 11 cases with advanced cervical dilation at least 3 cm, including two cases near fully dilated before the operation, underwent successful ECC without procedure-related rupture of membrane, suggesting that the amnioreduction may be a feasible option before ECC in such conditions.

In view of the effectiveness of ECC, the prolongation of pregnancy and neonatal survival are both important points. Even with low and very low quality of evidence, the benefits of ECC have been confirmed by three comprehensive reviews [11, 12, 14], presenting the favorable prolongation of pregnancy from 4–5 weeks to 47 days, 2-fold reduction of preterm birth before 34 gestational weeks, later gestational age at delivery, and lower risks of fetal loss, very preterm birth and neonatal death. However, the pregnancy outcomes after ECC with amnioreduction have not been fully elucidated due to the lack of universal acceptance among clinicians and the rarity of valid control cases. Locatelli A. et al. have reported 16 patients with cervical dilation (2–5 cm) and prolapse of fetal membranes, including seven cases with ECC and nine cases with ECC and amnioreduction, and found that with the comparable prolongation of pregnancy, the amnioreduction before ECC is associated with a lower rate of delivery before 32 weeks and neonatal morbidity [23]. One recent study by Cakiroglu Y. et al. found that ECC yielded no different outcomes for prolongation of pregnancy and live birth rate after comparing between amnioreduction group and non-amnioreduction group [22]. Another compared study from Japan also showed similar pregnancy outcomes after ECC between eight patients with amnioreduction and nine cases without amnioreduction, even though the size of forewater detected by ultrasound was larger in amnioreduction group [24]. Furthermore, our study comprised relative more cases who underwent ECC with amnioreduction (*n* = 16) or no-amnioreduction (*n* = 30), and also found that there were no significant differences in prolongation of pregnancy, gestational weeks at delivery, live birth rate and neonatal survival between the two groups, nevertheless, the mean of cervical dilation in amnioreduction cases was significantly larger than that in non-amnioreduction cases, suggesting that amnioreduction may be a safe and feasible option before ECC.

The major limitation of our study was the lack of testing intra-amniotic infection or inflammation from the amniotic fluid withdrawn among cases with amnioreduction. The rates of intra-amniotic infection and inflammation determined by amniocentesis have been reported as high as 52% and 81%, respectively [28, 29]. However, a recent observational study found that antibiotics could effectively eradicate 75% of intra-amniotic inflammation and 60% of infection [30]. In our study, the broad-spectrum antibiotics were routinely administrated to all the cases during the perioperative period, and the rates of clinical and histologic chorioamnionitis after ECC were not significantly different between the two groups. Moreover, the benefits of excluding some cases of sub-clinical chorioamnionitis before ECC and increasing the risk of infection for exposure to bulging membranes to the vaginal bacteria when awaiting the results were hard to balance. Another major limitation was the retrospective and non-randomized control study with a relatively small sample size, which inevitably comprised unmatched baseline variables (such as maternal age and cervical dilation before operation) and also can lead a potential bias of selection and limit the statistical power. According to the sample size calculation (Table 3) based on the present results, the current sample size may not have statistical power to detect the difference in most parameters of pregnancy outcomes between two groups and this suggested that the definitive recommendation could not be drawn from the current data. Other limitations included different devices such as moist gauze and inflated balloon, or Foley catheter used during the procedure at the discretion of operators, no uniformity of perioperative tocolytic treatment strategies based on individual basis, and lack of long-term outcomes of infants. Therefore, further prospective studies with proper design and larger sample size to explore this issue are desirable.

**Table 3:** Sample size calculation based on the present results.

Variables	Effective size	Alpha (one-tailed)	Power	Calculated sample size per group
Prolongation of pregnancy (days)	0.54	0.05	0.8	44
Preterm premature rupture of membranes	/	0.05	0.8	951
Miscarriage or abortion <24 weeks	/	0.05	0.8	129
Delivery 24-27^+6^ weeks	/	0.05	0.8	129
Delivery ≥28 weeks	/	0.05	0.8	10
Delivery ≥32 weeks	/	0.05	0.8	96
Live birth	/	0.05	0.8	270

In summary, although with larger cervical dilation, patients who underwent ECC with amnioreduction got the acceptable pregnancy outcomes when compared with cases who had relatively smaller cervical dilation without amnioreduction. Even definitive recommendations could not be drawn from this limited sample size, these findings suggested that amnioreduction may be a safe and feasible option to be applied before ECC, especially for those with advanced cervical dilation and bulging membranes with high pressure. Further prospective studies with larger sample to evaluate the benefits of amnioreduction before ECC will be needed to confirm our findings.

## Data Availability

The original contributions presented in the study are included in the article/Supplementary Material, further inquiries can be directed to the corresponding author/s.

## Supplementary Material

The Supplementary Material for this article can be found online at: https://www.frontiersin.org/articles/10.3389/fsurg.2022.928322/ full#supplementary-material.
