# Supplementary material for: The Role of Amnioreduction in Emergency Cervical Cerclage with Bulging Membranes: A Retrospective Comparative Study
Source: Front Surg. 2022 Jul 12;9:928322. doi: 10.3389/fsurg.2022.928322 (PMC9314748; doi:10.3389/fsurg.2022.928322)
Supplement: Supplementary file 1 [file Table_1_v1.docx]

**Supplementary Table S1 Clinical features and outcomes of patients in the amnioreduction group**

| No. | age | G | P | Cervical dilation (cm) | GA at cerclage (weeks + days) | Symptoms | DVP of amniotic fluid before operation (mm) | Volume of amniotic fluid withdraw | DP of amniotic fluid after operation(mm) | Prolongation of pregnancy (days) | Post-operation complication | GA at delivery (weeks + days) | Mode of delivery | Neonatal birthweight | Neonatal Apgar scores-1 min | Neonatal Apgar scores-5 min | Acute histologic chorioamnionitis |  |
| --- | --- | --- | --- | --- | --- | --- | --- | --- | --- | --- | --- | --- | --- | --- | --- | --- | --- | --- |
| 1 | 31 | 1 | 0 | 2 | 16 | vaginal bleeding | 39 | 100 | 32 | 2 | Clinical chorioamnionitis | 16+2 | D&C | — | — | — | Yes |  |
| 2 | 34 | 5 | 3 | 6 | 19+5 | None | 40 | 80 | 38 | 15 | spontaneous miscarriage | 21+6 | VD | — | stillbirth | | N/A |  |
| 3 | 41 | 2 | 1 | 8 | 19+5 | vaginal bleeding | 45 | 60 | 40 | 28 | spontaneous miscarriage | 23+5 | VD | 550 | stillbirth | | N/A |  |
| 4 | 31 | 1 | 0 | 9 | 20+4 | vaginal bleeding | 46 | 100 | 32 | 9 | Clinical chorioamnionitis | 21+6 | VD | 500 | stillbirth | | Yes |  |
| 5 | 29 | 1 | 0 | 2 | 21+6 | None | 42 | 80 | 36 | 3 | pPROM | 22+2 | VD | — | stillbirth | | Yes |  |
| 6 | 36 | 3 | 0 | 3 | 22+1 | None | 57 | 160 | 40 | 88 | preterm labor | 34+5 | CS | 2300 | 10 | 10 | No |  |
| 7 | 30 | 1 | 0 | 6 | 22+1 | Increased vaginal discharg | 70 | 230 | 44 | 15 | pPROM | 24+2 | VD | 690 | stillbirth | | Yes |  |
| 8 | 24 | 2 | 0 | 3 | 23 | vaginal bleeding | 40 | 50 | 35 | 5 | spontaneous miscarriage | 23+5 | VD | 660 | stillbirth | | N/A |  |
| 9 | 35 | 2 | 0 | 2 | 24+1 | None | 38 | 100 | 30 | 27 | preterm labor | 28 | VD | 1000 | 10 | 10 | No |  |
| 10 | 32 | 1 | 0 | 5 | 24+3 | Sensation of pressure | 52 | 200 | 25 | 21 | preterm labor | 27+3 | VD | 1020 | 8 | 9 | Yes |  |
| 11 | 36 | 4 | 2 | 3 | 25+1 | Increased vaginal discharge | 71 | 100 | 39 | 14 | pPROM | 27+1 | VD | 1120 | 4 | 9 | Yes |  |
| 12 | 33 | 2 | 1 | 6 | 25+6 | vaginal bleeding | 46 | 250 | 41 | 14 | preterm labor | 27+6 | VD | 1150 | 9 | 9 | Yes |  |
| 13 | 31 | 1 | 0 | 6 | 25+6 | vaginal bleeding | 43 | 100 | 40 | 47 | pPROM | 32+4 | CS | 1700 | 9 | 10 | Yes |  |
| 14 | 37 | 3 | 0 | 3 | 25+6 | Sensation of pressure | 48 | 100 | 35 | 14 | preterm labor | 27+6 | VD | 1200 | 10 | 10 | No |  |
| 15 | 38 | 1 | 0 | 3 | 25+2 | None | 67 | 170 | 52 | 33 | preterm labor | 30 | VD | 1750 | 10 | 10 | No |  |
| 16 | 26 | 2 | 0 | 5 | 24+2 | vaginal bleeding | 38 | 70 | 31 | 5 | pPROM | 25 | VD | 740 | 9 | 9 | Yes |  |

GA, Gestational age; DVP, Deepest vertical pocket; pPROM, Preterm premature rupture of membranes; VD, Vaginal delivery; CS, Caesarean section; D&C, Dilation and curettage; N/A: Not assessed
