# Supplementary material for: The Role of Amnioreduction in Emergency Cervical Cerclage with Bulging Membranes: A Retrospective Comparative Study
Source: Front Surg. 2022 Jul 12;9:928322. doi: 10.3389/fsurg.2022.928322 (PMC9314748; doi:10.3389/fsurg.2022.928322)
Supplement: Supplementary file 2 [file Table_2_v1.docx]

**Supplemental Table S2 Clinical features and outcomes of patients in the non-amnioreduction group**

| No. | age | G | P | Cervical dilation (cm) | GA at cerclage (weeks + days) | Symptoms | DVP of amniotic fluid before operation(mm) | Prolongation of pregnancy (days) | Post-operation complication | GA at delivery (weeks+days) | Mode of delivery | Neonatal birthweight | Neonatal Apgar scores-1min | | Neonatal Apgar scores-5min | Acute histologic chorioamnionitis |  |
| --- | --- | --- | --- | --- | --- | --- | --- | --- | --- | --- | --- | --- | --- | --- | --- | --- | --- |
| 1 | 22 | 3 | 0 | 1 | 15+5 | None | 34 | 165 | None | 39+2 | VD | 3200 | 10 | | 10 | N/A |  |
| 2 | 32 | 1 | 0 | 3 | 16+5 | Sensation of pressure | 38 | 97 | preterm labor | 30+4 | VD | 1800 | 10 | 10 | | Yes |  |
| 3 | 27 | 2 | 0 | 2 | 17+6 | None | 30 | 13 | spontaneous miscarriage | 19+5 | VD | — | stillbirth | | | N/A |  |
| 4 | 26 | 2 | 0 | 1 | 18+1 | vaginal bleeding | 48 | 11 | pPROM | 19+5 | VD | — | stillbirth | | | Yes |  |
| 5 | 26 | 1 | 0 | 6 | 19+4 | vaginal bleeding | 26 | 1 | spontaneous miscarriage | 19+5 | VD | — | stillbirth | | | N/A |  |
| 6 | 34 | 4 | 1 | 3 | 19+5 | None | 41 | 0 | pPROM during operation | 19+5 | VD | 410 | stillbirth | | | N/A |  |
| 7 | 36 | 6 | 1 | 2 | 20+2 | Increased vaginal discharge | 50 | 3 | pPROM | 20+5 | VD | — | stillbirth | | | Yes |  |
| 8 | 28 | 3 | 0 | 4 | 20+4 | None | 31 | 4 | spontaneous miscarriage | 21+1 | VD | — | stillbirth | | | Yes |  |
| 9 | 27 | 2 | 0 | 1 | 20+5 | vaginal bleeding | 40 | 7 | spontaneous miscarriage | 21+5 | VD | 490 | stillbirth | | | N/A |  |
| 10 | 28 | 1 | 0 | 3 | 20+6 | None | 32 | 36 | pPROM | 26 | VD | 900 | 5 | 10 | | Yes |  |
| 11 | 32 | 2 | 1 | 2 | 21+5 | vaginal bleeding | 52 | 49 | preterm labor | 28+5 | VD | 1320 | 5 | 10 | | Yes |  |
| 12 | 37 | 2 | 1 | 2 | 22+4 | None | 38 | 21 | pPROM | 25+4 | VD | 890 | 10 | 10 | | Yes |  |
| 13 | 33 | 2 | 1 | 3 | 23+1 | None | 40 | 7 | spontaneous miscarriage | 24+1 | VD | 600 | stillbirth | | | Yes |  |
| 14 | 25 | 1 | 0 | 2 | 23+5 | None | 54 | 42 | preterm labor | 29+5 | VD | 1350 | 10 | 10 | | No |  |
| 15 | 29 | 2 | 1 | 1 | 23+5 | None | 49 | 97 | None | 37+4 | VD | 3600 | 10 | | 10 | No |  |
| 16 | 29 | 3 | 0 | 1 | 23+6 | vaginal bleeding | 41 | 11 | Clinical chorioamnionitis | 25+3 | VD | 690 | stillbirth | | | Yes |  |
| 17 | 28 | 3 | 1 | 2 | 24+1 | Sensation of pressure | 45 | 73 | pPROM | 34+4 | VD | 2800 | 10 | | 10 | No |  |
| 18 | 29 | 1 | 0 | 3 | 24+3 | None | 47 | 98 | None | 38+3 | CS | 3050 | 10 | 10 | | N/A |  |
| 19 | 22 | 1 | 0 | 1 | 24+4 | None | 51 | 107 | None | 39+6 | VD | 3450 | 10 | | 10 | N/A |  |
| 20 | 37 | 5 | 1 | 1 | 24+4 | None | 35 | 5 | pPROM | 25+2 | VD | 560 | stillbirth | | | Yes |  |
| 21 | 30 | 1 | 0 | 4 | 24+5 | vaginal bleeding | 33 | 24 | pPROM | 28+1 | VD | 1150 | 8 | | 10 | Yes |  |
| 22 | 34 | 4 | 0 | 1 | 24+5 | None | 56 | 21 | pPROM, infection | 27+5 | CS | 1000 | 10 | | 10 | Yes |  |
| 23 | 27 | 2 | 0 | 1 | 25+1 | None | 42 | 63 | preterm labor | 34+1 | VD | 2100 | 10 | 10 | | Yes |  |
| 24 | 37 | 4 | 1 | 2 | 25+3 | None | 41 | 60 | preterm labor | 34 | CS | 1800 | 10 | | 10 | No |  |
| 25 | 28 | 3 | 1 | 3 | 25+5 | None | 35 | 17 | preterm labor | 28+1 | VD | 1340 | 8 | | 10 | Yes |  |
| 26 | 29 | 1 | 0 | 3 | 26+2 | vaginal bleeding | 48 | 2 | pPROM | 26+4 | VD | 850 | 8 | | 9 | No |  |
| 27 | 27 | 1 | 0 | 3 | 26+5 | vaginal bleeding | 43 | 31 | pPROM | 31+1 | VD | 1250 | 10 | 10 | | No |  |
| 28 | 30 | 3 | 1 | 3 | 27+1 | vaginal bleeding | 46 | 29 | preterm labor | 31+2 | VD | 1850 | 10 | | 10 | No |  |
| 29 | 31 | 2 | 0 | 1 | 27+6 | None | 31 | 22 | pPROM | 32+1 | CS | 1550 | 10 | | 10 | No |  |
| 30 | 22 | 2 | 0 | 1 | 26 | None | 36 | 38 | preterm labor | 32+3 | VD | 1600 | 10 | | 10 | No |  |

GA, Gestational age; DVP, Deepest vertical pocket; pPROM, Preterm premature rupture of membranes; VD, Vaginal delivery; CS, Caesarean section; N/A: Not assessed
